# Effects of stem cell factor in follicular fluid and granulosa cells on oocyte maturity and clinical pregnancy

**DOI:** 10.1097/MD.0000000000036749

**Published:** 2023-12-29

**Authors:** Xu Wang, Lixiang Zhou, Anli Xu, Dunzhu NIMA, Zhaomei Dong

**Affiliations:** a Department of Obstetrics and Gynecology, People’s Hospital of Leshan, Leshan, China; b Department of Gynecology, People’s Hospital of Anshun, Anshun, China; c Department of Reproductive Medicine, The First Affiliated Hospital of Dali University, Dali, China; d Clinical Lab, Tibet Autonomous Region People’s Hospital, Lhasa, China.

**Keywords:** clinical pregnancy, follicular fluid, granulosa cells, MII oocyte, stem cell factor

## Abstract

Stem cell factor (SCF) is implicated in cell growth, proliferation, differentiation, migration, and apoptosis. SCF in follicular fluid (FF) and granulosa cells (GCs) plays a key role in oocyte maturation and clinical pregnancy; however, the exact mechanism is unclear. We aimed to investigate SCF potential in predicting oocyte maturity and clinical pregnancy. We collected 60 FF and 60 GCs samples from different patients with infertility. Real-time polymerase chain reaction and cellular immunofluorescence analyses were used to quantitatively and qualitatively determine SCF concentration in GCs; enzyme-linked immunosorbent assay was used to determine SCF concentration in FF. GC and FF SCF concentrations were positively correlated with metaphase (M)II oocyte proportion and clinical pregnancy (*R* = 0.280, 0.735 vs *R* = 0.257, 0.354). SCF concentrations in GCs were significantly higher in the clinical pregnancy group than in the nonclinical pregnancy group. Immunofluorescence analysis showed that SCF expression was higher in the clinical pregnancy and high-MII -oocyte proportion groups. Receiver operating characteristic curve analysis showed that combined SCF and serum anti-Müllerian hormone levels could predict oocyte maturity and clinical pregnancy better than either of these factors alone. SCF concentration in GCs and FF can serve as a predictor of oocyte maturity and clinical pregnancy.

## 1. Introduction

Stem cell factor (SCF) is a ligand of receptor tyrosine kinases and it binds specifically to type III tyrosine kinase receptor (c-Kit); hence, it is also known as Kit ligand.^[1]^ Receptor tyrosine kinases are the main players in signal transduction in cells during normal regeneration and tumor progression. Their activities are regulated by the availability and potency of their cognate ligands, making them valuable treatment targets and research subjects in various diseases.^[2]^ The c-Kit signaling pathway promotes cell proliferation, differentiation, and survival. c-Kit mutations have been associated with several human malignant tumors, such as gastrointestinal stromal tumors,^[3]^ liver cancer,^[4]^ prostate cancer,^[5]^ and rectal cancer.^[6]^Within the reproductive system, Follicular fluid (FF) forms the pre-ovulation microenvironment necessary for oocyte growth and maturation, from single biochemical markers to metabolomics.^[7,8]^ SCF in FF is mainly secreted by granulosa cells (GCs). Ligand–receptor interactions establish communication between oocytes and GCs, participate in the development and growth of primordial follicles and the appearance of preovulatory dominant follicles,^[1,9]^ and play an important role in early embryonic development and blastocyst formation.^[10,11]^

Mouse and rat ovaries have been used to detect the expression of phosphoinositide 3 kinase/serine-threonine protein kinase (PI3K/AKT) in oocytes. Previous studies found that this pathway was regulated by SCF in GCs, which was vital for early follicular development, and its blockage resulted in impaired follicular development and infertility.^[12,13]^ In vitro co-culture of mouse oocytes and SCF showed that SCF substantially promoted the extrusion of the first polar body in preovulatory oocytes and promoted follicle development and maturation. In vivo inhibition of SCF/c-Kit signaling impaired primordial follicle growth and development, FF formation in preantral follicles, and the second stage of preovulatory follicle maturation and ovulation.^[14]^ These findings are of great significance in identifying and regulating new pathways or targets for primordial follicle activation and finding new therapeutic approaches for infertility.^[15]^ Presently, the regulatory mechanism of SCF in human follicular development remains unclear. Serum anti-Müllerian hormone (AMH) level is a marker of ovarian reserve. Although many indicators, such as antral follicle count, patient age, serum AMH levels, and basal ovarian volume, are considered when assessing ovarian reserve, these indicators are less accurate to evaluate follicular development. When researchers simultaneously investigated the effects of SCF and AMH on follicular development, they found that AMH downregulated SCF mRNA and protein expression in human GCs in a dose-dependent manner through the cAMP/PKA pathway, which increased *SCF* transcription.^[16]^ AMH and SCF are key factors in follicle regeneration and development, and the balance between AMH and SCF plays a crucial role in follicle development and maturation.^[17]^

Due to the general decline in fertility potential, more and more infertility patients are turning to assisted reproductive technology (ART) to help them conceive. With the development of ART, the techniques for evaluating oocyte and embryo quality have been continuously innovated and improved, but these evaluation methods have certain limitations. To date, determining embryo quality with a highly accurate noninvasive quantitative embryo assessment technique for pregnancy has been a difficult goal to achieve.

**Figure 1.**
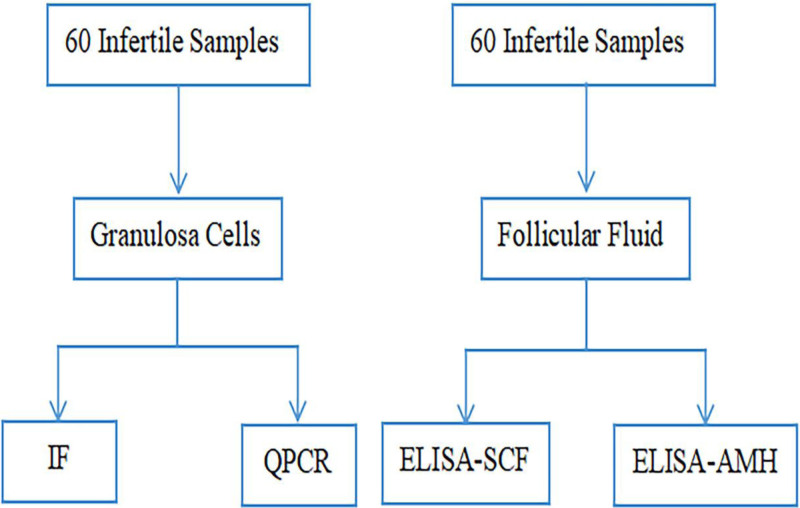
Overall analysis workflow. ELISA = enzyme-linked immunosorbent assay, IF = immunofluorescence, qPCR = real-time polymerase chain reaction.

In this study, we aimed to explore the relationship between different SCF contents in GCs and FF and to investigate SCF’s effects on oocyte maturity and clinical pregnancy. We further aimed to test our hypothesis that SCF level could serve as a biomarker to evaluate oocyte maturity and its potential clinical pregnancy rate to further improve the clinical pregnancy rate in patients with infertility. The workflow of this study is shown in Figure 1.

## 2. Materials and methods

### 2.1. Patients

Between January 2021 and August 2022, 60 patients with infertility undergoing in vitro fertilization-embryo transfer (IVF-ET) at our reproductive center were selected, and FF and GCs were collected during oocyte retrieval. All patients underwent ovarian stimulation using the long protocol of the follicular phase. The inclusion criteria included no history of polycystic ovary syndrome or premature ovarian failure, no more than two previous abortions, body mass index between 15 and 35 kg/m^2^, basal follicle-stimulating hormone (FSH) ≤ 15 IU/L, and no family history of genetic or chromosomal disease. Patients with a history of ovarian endometriomas, ovarian surgery, intrauterine adhesions, congenital uterine malformations, thyroid dysfunction, repeated graft failure (no pregnancy after three or more cycles), chromosomal abnormalities, acquired or hereditary thrombosis, chemotherapy or radiation therapy, acute infections, or newly diagnosed chronic infectious diseases were excluded. All relevant clinical data of the enrolled patients were recorded.

### 2.2. Controlled ovarian stimulation

Long-acting gonadotropin-releasing hormone agonist (3.75 mg) was administered on the 2nd to 4th day of menstruation to reduce the pituitary gland activity by desensitization. After 28 days, we checked the sex hormone levels and measured and recorded the follicle diameter using gynecological ultrasound to evaluate whether the standard of reduction was reached. The criteria used by our hospital’s reproductive center were as follows: estradiol < 50 pg/mL, luteinizing hormone < 5 IU/L, FSH < 5 IU/L, progestin (P) < 0.5 pg/mL, maximum follicle diameter < 10 mm, endometrial thickness < 5 mm, and absence of functional cysts. To promote follicular development, exogenous gonadotropin administration was started using either human menotropin gonadotrophin or recombinant human FSH (rFSH) according to the patient’s age and follicle growth. During the treatment, follicular growth, endometrial thickness, and morphology of the patients were regularly evaluated using B-ultrasound, and sex hormone levels were measured to adjust the dosage of gonadotropin in time. Gonadotropin administration was discontinued when the patient had at least two dominant follicles ≥ 18 mm in diameter, three dominant follicles ≥ 17 mm in diameter, or four dominant follicles ≥ 16 mm in diameter in both ovaries. Recombinant human chorionic gonadotropin (rhCG) (250 µg or rhCG 250 µg + human chorionic gonadotropin [hCG] 2000 IU) was injected at night on the same day. Transvaginal ultrasound-guided oocyte retrieval was performed 36 to 38 hours after trigger, and IVF was performed 4 to 6 hours after oocyte retrieval. The patients were prepared for transplantation based on conventional luteal support.

### 2.3. Collection of FF and determination of SCF and AMH concentrations

On the day of oocyte retrieval, all visible follicles (≥16 mm) were aspirated without washing. After oocyte retrieval, any unclear FF or obvious bloodstained FF samples were discarded. Clear FF samples without blood contamination were collected in a sterile test tube, labeled, and immediately sent to the laboratory for centrifugation at 3000 rpm in 4 °C for 10 minutes. The supernatant was aliquoted and stored at −80 °C until testing.

A commercially available enzyme-linked immunosorbent assay kit (Elabscience, Wuhan, China) was used to measure the concentrations of SCF and AMH in each sample.

### 2.4. GC collection, cellular immunofluorescence (IF) analysis, and real-time polymerase chain reaction (qPCR)

On the day of oocyte retrieval, all visible follicles (≥16 mm) were aspirated without washing. After oocyte retrieval, all GCs in the FF were extracted and separated through human lymphocyte separation medium (Tianjin Haoyang Biological Manufacture Co., Tianjin, China), and GC suspensions were prepared for qPCR and IF experiments.

An RNeasy total RNA extraction kit (Tiangen, Beijing, China) was used to extract total RNA from GCs. After determining the concentration, purity, and integrity of the extracted total RNA, a Super cDNA First-Strand Synthesis Reverse Transcription Kit (CWBiotech, Beijing, China) was used for the reverse transcription step to synthesize cDNA, which was stored at −80 °C until qPCR analysis. The reference gene *GAPDH* for the PCR primers and the primer sequences for the target gene *SCF* are listed in Table 1. Three compound wells were set for each sample and placed in an ABI Step One Plus qPCR instrument. The reaction conditions were 95 °C for 10 minutes, followed by 40 cycles of 10 seconds at 95 °C, 30 seconds at 60 °C, and 32 seconds at 72 °C.

**Table 1:** Primer sequences for the reference gene GAPDH and the target gene SCF.

Variable	Forward primer	Reverse primer
GAPDH	AGAAGGCTGGGGCTCATTTG	AGGGGCCATCCACAGTCTTC
SCF	CAGAGTCAGTGTCACAAAACCATT	TTGGCCTTCCTATTACTGCTACTG

SCF = stem cell factor.

The resulting cell suspension was plated on polylysine-treated coverslips, and after cell fixation, cell permeabilization, blocking, antibody incubation, and staining, the images were observed using an inverted fluorescence microscope (Olympus, Beijing, China).

### 2.5. Statistical analysis

All data are expressed as the mean ± standard deviation ($\bar{x}\pm s$), and the *t* test was used to compare the data between the two groups. For the comparison of rates in the data, the chi-square test or Fisher exact probability method was used for analysis according to the different sample sizes included in the groups. Spearman correlation test was used to analyze the correlation between SCF levels in GCs or FF and oocyte maturity and clinical pregnancy. Adobe Photoshop 2022 was used to analyze and process IF images. A receiver operating characteristic (ROC) curve was used to estimate the predictive value of SCF level for oocyte maturity and clinical pregnancy rate. SPSS 27.0 statistical software was used to analyze and process all the collected data. *P* < .05 was considered statistically significant.

## 3. Results

### 3.1. Relationship between SCF concentration in GCs and oocyte maturity and pregnancy

Spearman correlation analysis was conducted to evaluate the relationship of the *SCF* mRNA levels in GCs with metaphase II (MII) oocyte proportion and clinical pregnancy. *SCF* mRNA level was significantly correlated with MII oocyte proportion (*R* = 0.280, *P* = .030) and clinical pregnancy (*R* = 0.735, *P* < .001) (Table 2).

**Table 2:** Correlation of SCF mRNA level in GCs with oocyte maturity and pregnancy.

Indicator	SCF mRNA level (ng/mL)	
	*R*	*P* value
Proportion of MII oocytes (%)	0.280*	.030
Pregnancy	0.735***	<.001

GCs = granulosa cells, MII = metaphase II, SCF = stem cell factor.

* *P* < .05.

*** *P* < .001.

Based on the Vienna Consensus^[18]^ on ART, we defined the proportion of MII oocytes ≥ 75% as high proportion value of MII oocytes. Accordingly, the patients were divided into groups with high proportion of MII oocytes (n = 52) and low proportion of MII oocytes (n = 8). The differences in each index and *SCF* mRNA levels in GCs between the groups were compared (Table 3). Gonadotropin dosage, days of stimulation, Number of MII oocytes, and high-quality embryo proportion in the group with high proportion of MII oocytes were higher than those in the group with low proportion of MII oocytes, and the difference was significant (*P* < .05).

**Table 3:** Comparison of SCF mRNA levels in GCs and the indices in patients with high and low proportions of MII oocytes.

Indicator	Group with high proportion of MII oocytes (n = 52)	Group with low proportion of MII oocytes (n = 8)	*P* value
Age (yr)	31.35 ± 3.79	33.25 ± 2.77	.179
BMI (kg/m^2^)	22.91 ± 3.24	22.39 ± 2.93	.667
Duration of infertility (yr)	3.65 ± 2.12	4.63 ± 4.18	.538
Gn dosage (IU)	2417.07 ± 658.49	1876.56 ± 330.21	.002**
Days of stimulation (d)	11.19 ± 1.67	9.75 ± 1.17	.022*
PRL (ng/mL)	16.96 ± 7.07	17.08 ± 6.71	.964
Basal LH (IU/L)	5.84 ± 4.67	3.98 ± 1.07	.267
Basal FSH (IU/L)	6.82 ± 1.79	6.06 ± 0.99	.249
P (ng/mL)	0.37 ± 1.41	0.21 ± 0.17	.755
T (ng/mL)	0.22 ± 0.12	0.17 ± 0.10	.201
Basal E2 (pg/mL)	35.34 ± 26.46	33.23 ± 11.66	.826
Number of oocytes retrieved	12.85 ± 4.89	11.88 ± 4.73	.881
Number of MII oocytes	11.06 ± 4.56	6.75 ± 3.28	.013*
Number of high-quality embryos	3.65 ± 2.62	2.00 ± 2.45	.099
High-quality embryo proportion (%)	18 (34.62)	3 (37.50)	<.001***
Serum AMH level (ng/mL)	3.31 ± 2.05	2.34 ± 1.64	.205
SCF mRNA level (ng/mL)	46.83 ± 104.26	9.76 ± 15.85	.323

AMH = anti-Müllerian hormone, BMI = body mass index, E2 = estradiol, FSH = follicle-stimulating hormone, GCs = granulosa cells, Gn = gonadotropin, LH = luteinizing hormone, MII = metaphase II, P = progestin, PRL = prolactin, SCF = stem cell factor, T = testosterone.

* *P* < .05.

** *P* < .01.

*** *P* < .001.

A serum hCG level > 10 mIU/mL 14 days after embryo transfer was defined as biochemical pregnancy. At 30–35 days after embryo transfer, gestational sac and primitive heart tube pulsation were detected using ultrasound, and clinical pregnancy was defined. Patients were divided into clinical pregnancy (n = 19) and nonclinical pregnancy (n = 41) groups, according to whether they were clinically pregnant. The differences in each index and *SCF* mRNA levels in GCs between the groups were compared (Table 4). The number of high-quality embryos, proportion of high-quality embryos, and *SCF* mRNA levels were significantly higher in the clinical pregnancy group than in the nonclinical pregnancy group (*P* < .05).

**Table 4:** Comparisons of SCF mRNA levels in GCS and the indexes in patients with and without clinical pregnancy.

Indicator	Clinical pregnancy group (n = 19)	Nonclinical pregnancy group (n = 41)	*P* value
Age (yr)	30.53 ± 4.06	32.10 ± 3.48	.128
BMI (kg/m^2^)	22.02 ± 2.35	23.22 ± 3.50	.123
Duration of infertility (yr)	3.95 ± 3.15	3.71 ± 2.12	.729
Gn dosage (IU)	10.74 ± 1.15	11.12 ± 1.87	.413
Days of stimulation (d)	13.47 ± 4.03	11.49 ± 5.08	.140
PRL (ng/mL)	19.01 ± 6.95	16.03 ± 6.85	.124
Basal LH (IU/L)	7.20 ± 5.41	4.85 ± 3.68	.053
Basal FSH (IU/L)	6.74 ± 1.76	6.72 ± 1.73	.968
P (ng/mL)	0.74 ± 2.32	0.16 ± 0.12	.291
T (ng/mL)	0.24 ± 0.11	0.20 ± 0.11	.138
Basal E2 (pg/mL)	37.76 ± 30.24	33.80 ± 22.37	.571
Number of oocytes retrieved	13.47 ± 4.03	11.49 ± 5.08	.140
Number of MII oocytes	11.79 ± 3.61	9.88 ± 4.96	.138
Number of high-quality embryos	5.95 ± 1.81	2.27 ± 2.10	<.001***
High-quality embryo proportion (%)	6 (31.58)	8 (19.51)	<.001***
Serum AMH level (ng/mL)	3.08 ± 1.40	3.22 ± 2.31	.800
SCF mRNA level (ng/mL)	89.99 ± 103.06	19.59 ± 88.05	.008**

AMH = anti-Müllerian hormone, BMI = body mass index, E2 = estradiol, FSH = follicle-stimulating hormone, GCs = granulosa cells, Gn = gonadotropin, LH = luteinizing hormone, MII = metaphase II, P = progestin, PRL = prolactin, SCF = stem cell factor, T = testosterone.

* *P* < .05.

** *P* < .01.

*** *P* < .001.

### 3.2. IF-based detection of GC SCF expression

**Figure 2.**
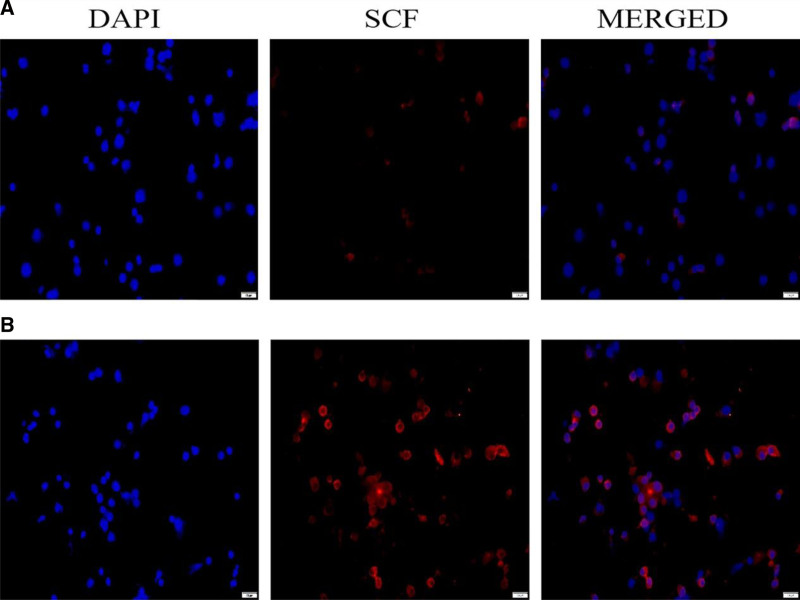
Difference in the expression levels of SCF protein in GCs between the groups with high and low proportions of MII oocytes. (A) Expression of SCF protein in GCs in the group with high proportion of MII oocytes. (B) Expression of SCF protein in GCs in the group with low proportion of MII oocytes. GCs = granulosa cells, MII = metaphase II, SCF = stem cell factor.

We further analyzed the differences in SCF protein expression levels in GCs between the groups with high and low proportions of MII oocytes and between the clinical and nonclinical pregnancy groups. SCF expression level in GCs in patients with a high proportion of MII oocytes was higher than that in patients with a low proportion of MII oocytes (Fig. 2).

**Figure 3.**
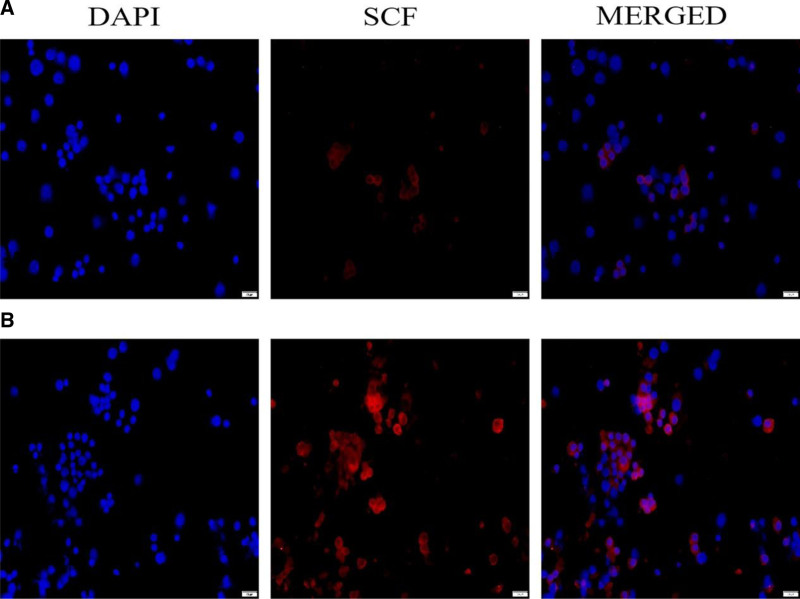
Difference in the expression levels of SCF protein in GCs between the clinical and nonclinical pregnancy groups. (A) Expression of SCF protein in GCs in the clinical pregnancy group. (b) Expression of SCF protein in GCs in the nonclinical pregnancy group. GCs = granulosa cells, SCF = stem cell factor.

Furthermore, the expression level of SCF protein in the clinical pregnancy group was higher than that in the nonclinical pregnancy group (Fig. 3).

### 3.3. ROC curve analysis of SCF in GCs and serum AMH for predicting oocyte maturity and clinical pregnancy

**Figure 4.**
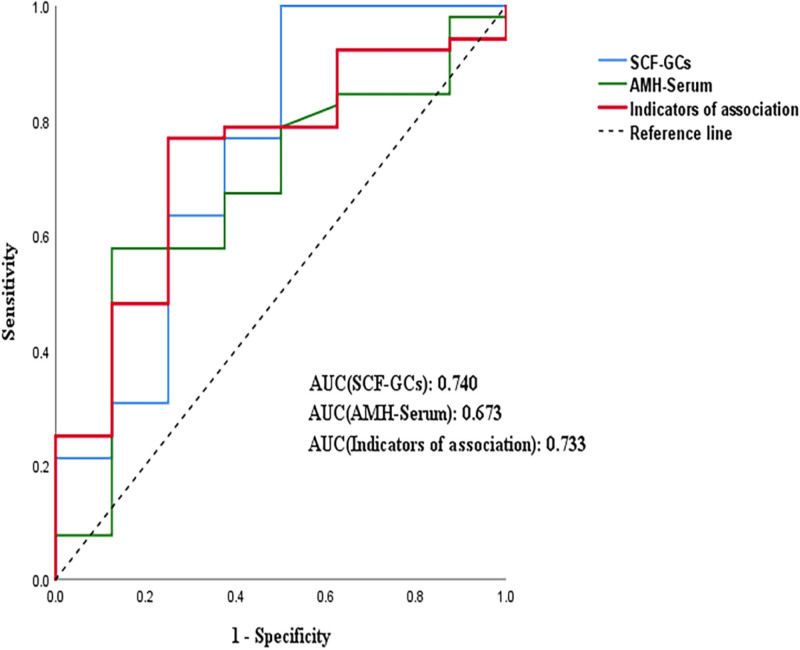
ROC curves show associations of SCF with high proportions of MII oocytes in GCs. GCs = granulosa cells, MII = metaphase II, ROC = receiver operating characteristic, SCF = stem cell factor.

A ROC curve of SCF in GCs combined with serum AMH levels was generated to evaluate its predictive value for oocyte maturity. After performing a binary logistic regression analysis, the combination of the above two indicators was entered into the regression curve. The area under the ROC curve (AUC) value of SCF in GCs was 0.740 (95% confidence interval [CI]: 0.552–0.959, *P* < .001). The AUC value of serum AMH was 0.673 (95% CI: 0.474–0.872, *P* = .639), and that of the combination of SCF in GCs and serum AMH was 0.733 (95% CI: 0.555–0.911, *P* < .001). The results showed that SCF in GCs alone and in combination with serum AMH had good predictive performance (Fig. 4).

**Figure 5.**
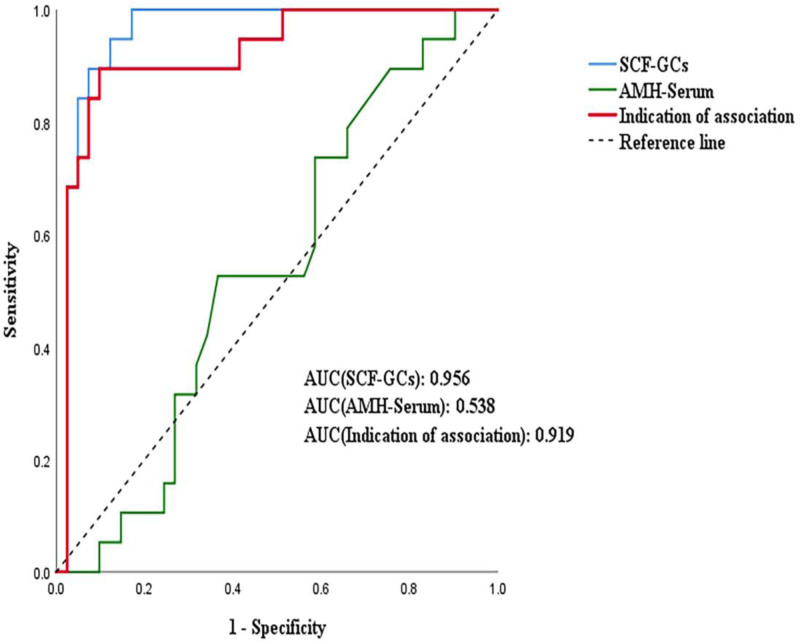
Pregnancy-related ROC curves in GCs. GCs = granulosa cells, ROC = receiver operating characteristic.

A ROC curve was generated by combining GC SCF and serum AMH levels to evaluate SCF’s predictive value for clinical pregnancy. After performing a binary logistic regression analysis, the combination of the two indicators was entered into the regression curve. The AUC value of SCF in GCs was 0.956 (95% CI: 0.903–1.000, *P* < .001), that of serum AMH was 0.538 (95% CI: 0.389–0.687, *P* = .639), and that of the combination of SCF in GCs and serum AMH was 0.919 (95% CI: 0.840–0.998, *P* < .001). The results showed that SCF in GCs alone and in combination with serum AMH had good predictive performance (Fig. 5).

### 3.4. Relationship between SCF concentration in FF with oocyte maturity and clinical pregnancy

To analyze the relationship between SCF level in FF and oocyte maturity and clinical pregnancy, Spearman correlation analysis was performed between SCF level in FF and MII oocyte proportion and clinical pregnancy. SCF was significantly associated with oocyte maturity (*R* = 0.257, *P* = .047) and clinical pregnancy (*R* = 0.354, *P* < .001) (Table 5).

**Table 5:** Correlation of SCF level in FF with oocyte maturity and clinical pregnancy.

Indicators	SCF (pg/mL)	
	*R*	*P* value
Proportion of MII oocytes (%)	0.257*	.047
Pregnancy	0.354***	<.001

FF = follicular fluid, MII = metaphase II, SCF = stem cell factor.

* *P* < .05.

*** *P* < .001.

To determine the correlation among FF AMH, FF SCF, and serum AMH levels, Spearman correlation analysis was used to analyze the concentration of AMH in FF, FF SCF, and serum AMH. The concentration of AMH in FF was significantly negatively correlated with the concentration of SCF in FF (*R* = −0.258, *P* = .046) and positively correlated with serum AMH concentration (*R* = 0.407, *P* = .001) (Table 6).

**Table 6:** Correlation analysis of FF AMH with FF SCF and serum AMH levels.

Indicators	Average AMH level in FF (ng/mL)	
	*R*	*P* value
Average SCF level in FF (pg/mL)	-0.258*	.046
Serum AMH level (ng/mL)	0.407**	.001

AMH = anti-Müllerian hormone, FF = follicular fluid, SCF = stem cell factor.

The differences in each index and FF SCF concentration between the groups with high (n = 39) and low proportions of MII oocytes (n = 21) were compared (Table 7). Number of MII oocytes, serum AMH, and average SCF levels in FF were higher in the group with high proportion of MII oocytes than in the group with low proportion of MII oocytes, and the difference was significant (*P* < .05).

**Table 7:** Comparisons of FF SCF levels with the indices in patients with high and low proportions of MII oocytes.

Indicator	Group with high proportion of MII oocytes (n = 39)	Group with low proportion of MII oocytes (n = 21)	*P* value
Age (yr)	29.97 ± 3.06	30.67 ± 3.28	.418
BMI (kg/m^2^)	22.68 ± 3.52	22.50 ± 2.87	.844
Duration of infertility (yr)	3.62 ± 1.73	4.29 ± 1.67	.153
Gn dosage (IU)	2117.63 ± 684.63	2175.60 ± 755.13	.764
Days of stimulation (d)	10.97 ± 1.41	10.95 ± 1.99	.960
PRL (ng/mL)	16.82 ± 5.51	17.55 ± 4.89	.615
Basal LH (IU/L)	5.93 ± 4.90	4.58 ± 3.10	.258
Basal FSH (IU/L)	6.81 ± 3.50	5.87 ± 1.91	.261
P (ng/mL)	0.21 ± 0.30	0.20 ± 0.14	.816
T (ng/mL)	0.33 ± 0.83	0.23 + 0.12	.563
Basal E2 (pg/mL)	32.91 ± 18.50	30.21 ± 17.74	.587
Number of oocytes retrieved	12.82 ± 4.32	12.48 ± 4.75	.777
Number of MII oocytes	11.05 ± 3.62	8.19 ± 3.61	.005**
Number of high-quality embryos	1.46 ± 0.82	1.43 ± 0.81	.882
High-quality embryo proportion (%)	10 (25.64)	7 (33.33)	.528
Serum AMH level (ng/mL)	4.32 ± 1.49	3.41 ± 1.64	.034*
Average SCF level in FF (pg/mL)	809.91 ± 178.96	671.86 ± 237.21	.014*
Average AMH level in FF (ng/mL)	1.22 ± 0.19	1.16 ± 0.24	.250

AMH = anti-Müllerian hormone, BMI = body mass index, E2 = estradiol, FF = follicular fluid, FSH = follicle-stimulating hormone, Gn = gonadotropin, LH = luteinizing hormone, MII = metaphase II, P = progestin, PRL = prolactin, SCF = stem cell factor, T = testosterone.

* *P* < .05.

** *P* < .01.

*** *P* < .001.

The differences in each index and FF SCF concentration between the clinical (n = 24) and nonclinical (n = 36) pregnancy groups were compared (Table 8). The number of oocytes retrieved, Number of MII oocytes, serum AMH, average SCF level in FF, and average AMH level in FF in the clinical pregnancy group were higher than those in the nonclinical pregnancy group. Age and duration of infertility in the clinical pregnancy group were lower than those in the nonclinical pregnancy group, and the differences were significant (*P* < .05).

**Table 8:** Comparisons of FF SCF levels and the indices in patients with and without clinical pregnancy.

Indicators	Clinical pregnancy group (n = 24)	Nonclinical pregnancy group (n = 36)	*P* value
Age (yr)	29.21 ± 3.12	30.89 ± 2.98	.040*
BMI (kg/m^2^)	22.48 ± 3.06	22.71 ± 3.46	.792
Duration of infertility (yr)	3.21 ± 1.67	4.28 ± 1.65	.018*
Gn dosage (IU)	1990.10 ± 569.88	2236.46 ± 773.08	.187
Days of stimulation (d)	10.54 ± 1.06	11.25 ± 1.86	.066
PRL (ng/mL)	16.60 ± 4.36	17.39 ± 5.84	.576
Basal LH (IU/L)	5.88 ± 4.57	5.17 ± 4.28	.543
Basal FSH (IU/L)	6.32 ± 1.98	6.58 ± 3.62	.747
P (ng/mL)	0.25 ± 0.38	0.18 ± 0.12	.283
T (ng/mL)	0.24 ± 0.10	0.33 + 0.86	.645
Basal E2 (pg/mL)	31.30 ± 16.52	32.40 ± 19.35	.821
Number of oocytes retrieved	14.13 ± 4.33	11.75 ± 4.31	.041*
Number of MII oocytes	12.04 ± 3.14	8.72 ± 3.73	.001**
Number of high-quality embryos	1.67 ± 0.64	1.31 ± 0.89	.091
High-quality embryo proportion (%)	10 (42.67)	7 (19.44)	.061
Serum AMH level (ng/mL)	4.50 ± 1.40	3.67 ± 1.65	.046*
Average SCF level in FF (pg/mL)	846.72 ± 189.99	704.84 ± 205.80	.009**
Average AMH level in FF (ng/mL)	1.27 ± 1.54	1.16 ± 0.23	031*

AMH = anti-Müllerian hormone, BMI = body mass index, E2 = estradiol, FF = follicular fluid, FSH = follicle-stimulating hormone, Gn = gonadotropin, LH = luteinizing hormone, MII = metaphase II, P = progestin, PRL = prolactin, SCF = stem cell factor, T = testosterone.

* *P* < .05.

** *P* < .01.

*** *P* < .001.

### 3.5. ROC curve of FF SCF and serum AMH levels for predicting oocyte maturity and clinical pregnancy

**Figure 6.**
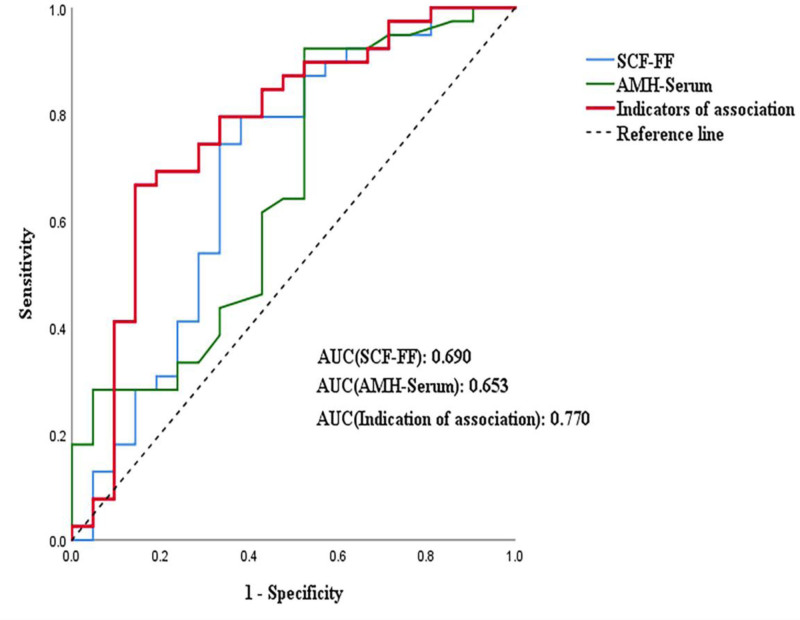
ROC curves associated with proportions of high MII oocytes in FF. FF = follicular fluid, MII = metaphase II, ROC = receiver operating characteristic.

A ROC curve was generated using the combination of FF SCF and serum AMH levels to estimate SCF’s predictive value for oocyte maturity. After performing a binary logistic regression analysis, the above combined indicators were entered into the regression curve. The AUC value of FF SCF was 0.690 (95% CI: 0.537–0.843, *P* = .016), that of serum AMH 0.653 (95% CI: 0.501–0.806, *P* = .052), and that of the both indicators combined 0.770 (95% CI: 0.635–0.903, *P* = .001). The combined AUC value was higher than that of FF SCF or serum AMH alone, confirming its better performance in predicting oocyte maturity (Fig. 6).

**Figure 7.**
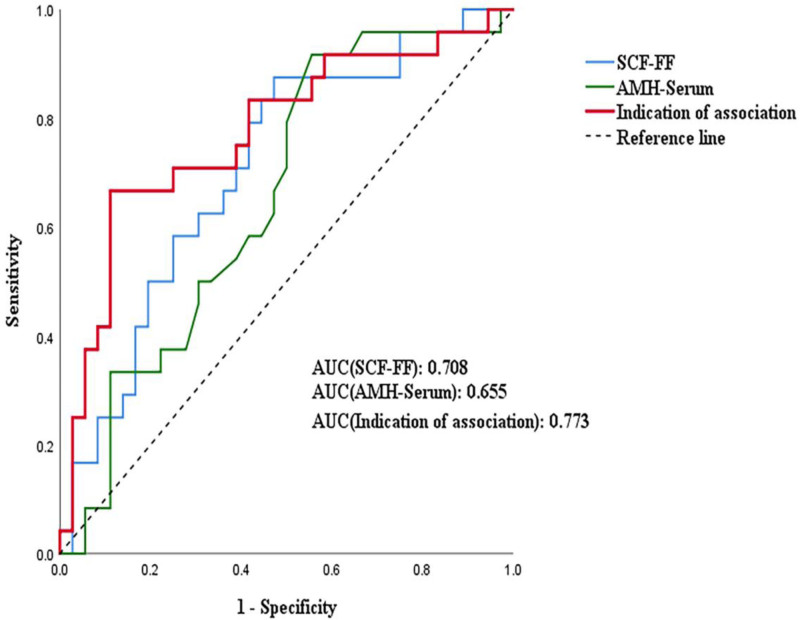
Pregnancy-related ROC curves in FF. FF = follicular fluid, ROC = receiver operating characteristic.

A ROC curve was generated using the combination of FF SCF and serum AMH levels to estimate its predictive value for clinical pregnancy. After performing a binary logistic regression analysis, the above combined indicators were entered into the regression curve. The AUC value of FF SCF was 0.708 (95% CI: 0.575–0.841, *P* = .007), that of serum AMH was 0.655 (95% CI: 0.516–0.794, *P* = .043), and m that of their combination was 0.773 (95% CI: 0.646–0.900, *P* < .001). The results showed that FF SCF alone could significantly predict the probability of pregnancy. When FF SCF and serum AMH were combined, the combined AUC value was larger than that of FF SCF or serum AMH alone, confirming its better performance in predicting clinical pregnancy (Fig. 7).

## 4. Discussion

In this study we addressed to the role of SCF in human follicular development. In addition to the accurate regulation of various hormones involved in follicle development,^[19,20]^ communication between GCs and oocytes is crucial for follicular development and oocyte maturation. Indeed, follicular development, oocyte maturation, cumulus expansion, and ovulation depend on the continuous bidirectional communication between GCs and oocytes.^[21,22]^ Some biological molecules in GCs may be able to accurately assess oocyte maturity and embryo quality and can even be used as diagnostic markers in ART.^[23]^ FF is where the oocytes grow and develop. Molecules secreted by the immune cells, including cytokines, proteins, electrolytes, reactive oxygen species, lipids, and vitamins contained in FF are often implicated in diseases of the female reproductive system. Follicles are major production sites of SCF, and the contributors to the concentration of SCF may impact follicular development. Changes in FF composition may affect the development, maturation, and ovulation of oocytes, thereby impairing female fertility.^[24]^ Therefore, accurately assessing the maturity of oocytes and improving the clinical pregnancy rate in patients undergoing ART are important aspects that need to be focused on. In this study, qPCR, IF, and enzyme-linked immunosorbent assay were used to investigate the effect of SCF on oocyte maturity and clinical pregnancy as well as to explore the predictors of oocyte maturity and clinical pregnancy in patients undergoing ART.

We observed that *SCF* mRNA levels in GCs were significantly positively correlated with the proportion of MII oocytes and clinical pregnancy. This means that *SCF* mRNA in GCs can be used to predict oocyte maturity and clinical pregnancy. Additionally, no significant difference in *SCF* mRNA levels in GCs was observed between the groups with high and low proportions of MII oocytes. However, this observation was the opposite to that of IF, in which the SCF expression level in GCs in patients who had a high MII oocyte proportion was higher than that in patients with low MII oocyte proportion. This implies that good oocyte maturity may be related to high SCF levels in GCs. Researchers examined SCF levels in FF and serum in patients with a poor response to IVF treatment.^[25]^ They observed a positive correlation between SCF expression levels in FF and serum and MII oocyte proportion, which was consistent with the results of our study. We also observed that the number of high-quality embryos, proportion of high-quality embryos, and *SCF* mRNA concentration were significantly higher in the clinical pregnancy group than in the nonclinical pregnancy group, which was consistent with the findings of Tan et al^[10]^ In IF analysis, SCF protein expression levels were higher in the clinical pregnancy group than in the nonclinical pregnancy group, consistent with the qPCR results. Based on the results of qPCR and IF, it can be concluded that SCF concentration may be an influencing factor in the clinical pregnancy group and pregnancy outcome.

We constructed a ROC curve of SCF in GCs combined with serum AMH and found that SCF alone had a good effect on predicting oocyte maturity and clinical pregnancy, although serum AMH is the currently recognized evaluation index. We also observed that combining these two factors had an advantageous predictive effect. The reason for the slightly lower AUC value obtained after combining the two factors may be the absence of any significant difference in serum AMH levels among the groups. However, this did not affect our exploration of the SCF prediction on oocyte maturity and clinical pregnancy.

The SCF level in FF was positively correlated with the proportion of MII oocytes and clinical pregnancy, suggesting that FF SCF level can be used to predict oocyte maturity and clinical pregnancy. The AMH level in FF was negatively correlated with the SCF level in FF and positively correlated with the level of AMH in the serum. This result provides evidence that AMH, as studied by Hu et al,^[16]^ may downregulate SCF expression in a dose-dependent manner. In our study, SCF expression in FF was higher in the groups with high proportion of MII oocytes and in the clinical pregnancy group. While exploring the relationship between SCF in FF and pregnancy outcomes, Hammadeh et al^[26]^ found no difference in FF SCF between 25 pregnant and 50 nonpregnant women in intracytoplasmic sperm injection (ICSI). Tan^[10]^ and Smikle et al^[27]^ analyzed the relationship between SCF in FF and clinical outcome. The authors found that high pregnancy was usually accompanied by a high SCF concentration, and the results were statistically significant. These contradictory results may be related to the fact that Hammadeh et al studied pregnancy outcomes in patients undergoing ICSI, whereas Tan and Smikle et al studied SCF in the FF of patients under IVF-ET. ICSI employs the artificial selection of sperm and complete fertilization, whereas IVF-ET excludes the factor of the artificial selection of sperm.

This study supports that SCF in FF is highly expressed during oocyte maturity and clinical pregnancy; therefore, it may predict oocyte maturity and clinical pregnancy. We performed a combined ROC curve analysis between SCF in FF and serum AMH. The combination of SCF and AMH in FF was more significant in predicting oocyte maturity and clinical pregnancy. However, FF from all follicles of the patient were collected together to detect the average SCF level in all FF of the patient and GCs collected from it. Therefore, the relationship between the SCF level of each follicle and the quality of each corresponding oocyte or embryo cannot be studied, and it is difficult to make a one-to-one comparative study. In addition, we did not evaluate the full spectrum of body mass index values, nor did we evaluate women with endometriosis and polycystic ovary syndrome. All of these variables are important predictors of oocyte maturity and clinical pregnancy rates.^[28,29]^In summary, our experimental analyses revealed that SCF might be a predictor of oocyte maturity and clinical pregnancy.

## 5. Conclusions

SCF may be a predictor of oocyte maturity and clinical pregnancy, and the combination of SCF and serum AMH levels can better predict oocyte maturity and clinical pregnancy. A significant negative correlation was observed between SCF and AMH in FF, suggesting that AMH downregulates SCF expression in a dose-dependent manner.

## Author contributions

**Conceptualization:** Zhaomei Dong.

**Data curation:** Lixiang Zhou.

**Methodology:** Xu Wang, Lixiang Zhou, Anli Xu.

**Resources:** Anli Xu, Zhaomei Dong.

**Software:** Xu Wang, Lixiang Zhou, Dunzhu NIMA.

**Writing – original draft:** Xu Wang.

**Writing – review & editing:** Zhaomei Dong.
